# Supplementary material for: Kombucha Reduces Hyperglycemia in Type 2 Diabetes of Mice by Regulating Gut Microbiota and Its Metabolites
Source: Foods. 2022 Mar 5;11(5):754. doi: 10.3390/foods11050754 (PMC8909623; doi:10.3390/foods11050754)
Supplement: Supplementary file 1 [file foods-11-00754-s001.zip › foods-1570200-supplementary.pdf]

Table S1. Standard for evaluation of pathology of colon.

Score	Colonic epithelium	Inflammatory reaction
0	Normal morphology	No inflammatory infiltration
1	Goblet cell injury	Inflammatory cell infiltration, infiltration around the recess
2	Large area injury of goblet cells and enlargement of intestinal epithelial cell gap	Inflammation invades mucosal muscular layer
3	Loss of crypt glands, damage to some villi and enlargement of intestinal epithelial cells	Invade the mucosal muscle layer more widely, mucosal edema appears
4	Large area loss of crypt glands, ulceration, massive destruction of villi	Invading submucosa

Table S2. Reaction system of reverse transcription.

Reagent	Dosage
dNTP Mix	4.0 μL
Primer Mix	2.0 μL
5×RT Buffer	4.0 μL
DTT	2.0 μL
HiFiScript	1.0 μL
RNA Template	1.0 μL
RNase-Free Water	6.0 μL

Table S3. Primer sequences.

Gene	Upper primer	Lower primer
IL-1β	CTGTGTCTTTCCCGTGGACC	CAGCTCA TATGGGTCCGACA
IL-6	AGAGGAGACTTCACAGAGGATACC	AATCAGAATTGCCATTGCACAAC
TNF-α	GACGTGGAACTGGCAGAAGAG	TCTGGAAGCCCCCCATCT
Muc2	ACCACCATTACCACCACCTCAG	CGATCACCACCATTGCCACTG
ZO-1	CCATCTTTGGACCGATTGCTG	TAATGCCCGAGCTCCGATG
Claudin-1	GGATGTCCTGCGTTTC	CACAGCCAAGACCCTC
Occludin	TTGGGAGCCTTGACATCTTGTTC	GCCATACATGTCATTGCTTGGTG
GPR41	AATCACAGAAACGGGGAAGCC	GTCTGGGGTCATTCTAATTGG
GPR43	GCTGGTGATGCTGGAAAGAAG	GGCATAACAGTGGAGACAAGAC
GADPH	TCCTGCACCACCAACTGCTTAG	CTTACTCCTTGGAGGCCATGT

Table S4. Reaction system of RT-qPCR.

Reagent	Dosage
cDNA Template	1.0 μL
Forward primer (10 μM)	1.0 μL
Reverse primer (10 μM)	1.0 μL
2 × qPCR SuperMix	10.0 μL
ddH_2O	7.0 μL
Total	20.0 μL

Table S5.Food intake and body weight correlation analysis.

		Body weight (g)
**Food intake (g/mouse/day)**	R	-0.927**
	P	0.00

Note: ***p*<0.01 indicates that there is a correlation between them.

Table S6. Effect of kombucha on the *α* diversity of gut microbiota in mice.

Group	Shannon	Simpson	Chao	ACE
NC	6.78±0.15	0.96±0.21	2558.77±140.11	2512.05±95.75
DC	6.48±0.11*	0.91±0.01*	2344.76±68.71*	2437.92±101.75*
MET	6.86±0.12#	0.98±0.01#	2687.67±57.75##	2766.77±88.25##
KT	6.87±0.23#	0.99±0.01#	2442.22±78.72#	2477.61±71.11#
TS	6.59±0.30	0.95±0.02	2402.75±61.15#	2462.30±63.15

Note: **p*<0.05, ***p*<0.01 vs. Normal; #*p*<0.05, ##*p*<0.01 vs. Model.

Table S7.LPS and IL-6 and TFN-*α* correlation analysis.

		TFN-*α* (pg/mL)	IL-6 (pg/mL)
**LPS (EU/L)**	R	0.974**	0.973**
	P	0.00	0.00

Note: ***p*<0.01 indicates that there is a correlation between them.

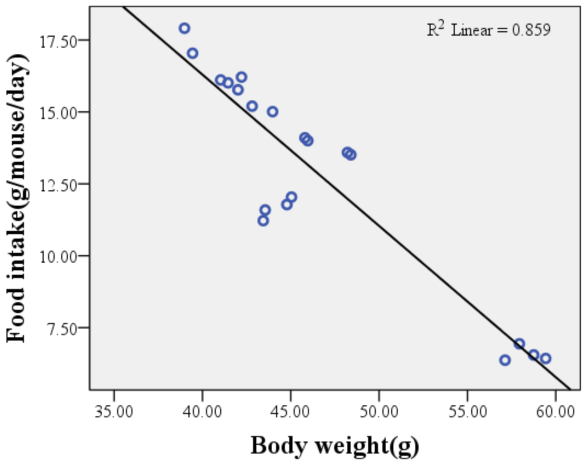

Figure S1.Correlation analysis of body weight and food intake.

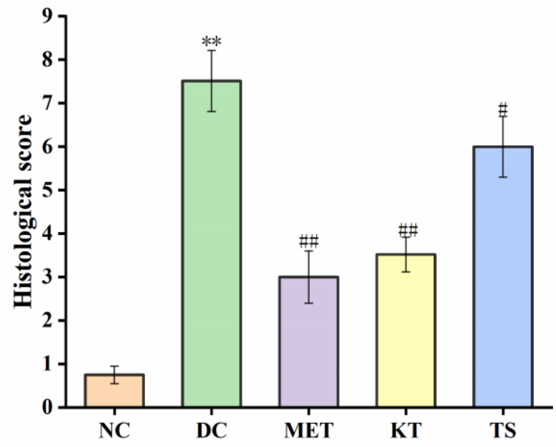

Figure S2.Colon histological score.
